# ELABELA attenuates deoxycorticosterone acetate/salt-induced hypertension and renal injury by inhibition of NADPH oxidase/ROS/NLRP3 inflammasome pathway

**DOI:** 10.1038/s41419-020-02912-0

**Published:** 2020-08-22

**Authors:** Zhida Chen, Chunying Wu, Yuting Liu, Haonan Li, Yeyan Zhu, Cailing Huang, Huangbo Lin, Qiao Qiao, Mengming Huang, Qing Zhu, Lei Wang

**Affiliations:** 1 School of Pharmaceutical Sciences, Guangzhou University of Chinese Medicine, Guangzhou, China; 2 Guangdong Metabolic Disease Research Center of Integrated Chinese and Western Medicine, Guangdong Pharmaceutical University, Guangzhou, China; 3 Department of Nephrology, the Second Affiliated Hospital, School of Medicine, Zhejiang University, Hangzhou, China

**Keywords:** Cell biology, Medical research

## Abstract

ELABELA (ELA), a 32-residue hormone peptide abundantly expressed in adult kidneys, has been identified as a novel endogenous ligand for APJ/Apelin receptor. The aim of this study was to investigate the role of ELA in deoxycorticosterone acetate (DOCA)/salt-induced hypertension and further explore the underlying mechanism. In DOCA/salt-treated rats, the mRNA level of ELA greatly decreased in the renal medulla. Next, overexpression of ELA in the kidney was found to attenuate DOCA/salt-induced hypertension and renal injury, including lower blood pressure, reversed glomerular morphological damage, decreased blood urea nitrogen (BUN), and blocked the accumulation of fibrotic markers. Mechanistically, ELA overexpression inhibited renal nicotinamide adenine dinucleotide phosphate (NADPH) oxidase activity and subsequent reactive oxygen species (ROS) production, thus resulted in the blockade of formation and activation of Nod-like receptor protein 3 (NLRP3) inflammasome. The inhibitory effects of ELA on Aldosterone-stimulated NADPH oxidase/ROS/NLRP3 inflammasome pathway were confirmed in the human renal tubular cells. Furthermore, our in vivo and in vitro results showed that the deficiency of the apelin receptor APJ did not influence the antihypertensive effect and blockage to NADPH oxidase/ROS/NLRP3 pathway of ELA. Moreover, in heterozygous ELA knockout mice (ELA^+/−^), the ELA deficiency remarkably accelerated the onset of DOCA/salt-induced hypertension. Our data demonstrate that ELA prevents DOCA/salt-induced hypertension by inhibiting NADPH oxidase/ROS/NLRP3 pathway in the kidney, which is APJ independent. Pharmacological targeting of ELA may serve as a novel therapeutic strategy for the treatment of hypertensive kidney disease.

## Introduction

Both human and animal studies demonstrate that high level of dietary salt contributes to the development of hypertension^1^. It is found that excessive salt intake leads to the imbalance of volume homeostasis and sodium reabsorption, which ultimately results in the elevation of blood pressure (BP)^2^. During this process, kidney is a major organ for BP homeostasis. Recently, there is a growing body of evidence suggesting that inflammation plays a crucial role in the progression of salt-sensitive hypertension as well as kidney dysfunction^3–5^. In experimental models, salt-sensitive hypertension are associated with the accumulation and activation of T cells in the kidney, which produces inflammatory cytokines including IFN-γ, IL-17, and TNF-α; results in sodium retention, vasoconstriction and enhanced oxidative stress; and then promotes hypertension, glomerular injury, renal fibrosis, and dysfunction^6^.

Inflammasomes are known as upstream machineries to trigger inflammation in response to danger signals from pathogens or damaged host^7,8^. Among them, Nod-like receptor protein 3 (NLRP3) inflammasome is best characterized, which consists of three major proteins: NLRP3, the adaptor protein ASC, and the effector caspase-1. Once activated, the inflammasome recruits these three components and cleaves pro-caspase-1 to an active form (cleavage caspase-1) which triggers proteolytic cleavage of pro-IL-1β and pro-IL-18 to mature and secreted forms^9,10^. NLRP3 inflammasome has been reported to play a fundamental role in the pathogenesis of salt-sensitive hypertension^4,7,11,12^. The mice with NLRP3 inflammasome deficiency failed to increase BP upon the DOCA/salt treatment^7,11^. It is known that there are several pathways to activate the NLRP3 inflammasome in response to diverse endogenous and exogenous danger signals, such as reactive oxygen species (ROS)^13–15^. Nicotinamide adenine dinucleotide phosphate (NADPH) oxidase is a key enzyme to generate superoxide intracellularly. The activation of NADPH oxidase is initiated by the assembly of cytosolic subunits, p22^phox^, and gp91^phox^ within the membrane while p47^phox^ and p67^phox^ in the cytoplasm. p22^phox^ and gp91^phox^ are found to help electron transfer from NADPH to oxygen molecules yielding O_2_^– 16^. p47^phox^ is able to interact with the cytoplasmic tail of p22^phox^ and p67^phox^ binds to gp91^phox^, resulting in the recruitment of related enzyme complex^17,18^. Previous studies show that NADPH oxidase inhibitor apocynin or deficiency of gp91^phox^ or p47^phox^ attenuates the hypertensive response in the DOCA/salt-treated mice^19,20^, demonstrating that NADPH oxidase activity contributes to DOCA/salt-induced hypertension. In addition, inhibition of the NADPH oxidase subunit gp91^phox^ or O_2_^–^ scavenging suppresses NLRP3 inflammasome activation and ultimately ameliorates renal injury^14,21^. Taken together, these findings suggest that the NADPH oxidase/ROS/NLRP3 pathway serves as a key pathogenic mechanism of salt-sensitive hypertension.

ELABELA/Toddler/Apela (ELA) is a 32-amino-acid peptide as a novel endogenous ligand for the APJ/Apelin receptor^22^. Previous studies demonstrate that ELA plays diverse biological functions in embryonic development^22–25^. Actually, the expression of ELA is also detected abundantly in adult kidneys^26,27^, especially in renal medulla^28^, suggesting that ELA may have important roles in the kidney. Recently, ELA and its shortest furin-cleaved fragment ELA11 are found to have protective effects on hypoxia–reperfusion (H/R) induced renal inflammation and fibrosis^27^. Another group reveals that ELA regulates water homeostasis by binding to the APJ receptor^26^. Furthermore, exogenous ELA treated in heart is found to exhibit antihypertensive effects in angiotensin II-infused mice, high salt fed Dahl salt-sensitive rats and rodent pulmonary arterial hypertension models^28–30^. However, whether ELA in kidney may exhibit antihypertensive and reno-protective actions in salt-sensitive hypertension remains unknown. The present study was aimed to investigate the roles of ELA in the regulation of BP and renal injury in DOCA/salt-treated animals as well as the potential molecular mechanism involved.

## Methods

### Animals

Male Sprague–Dawley (SD) rats, C57BL/6 mice, and heterozygous ELA knockout C57BL/6 mice (ELA^+/−^) were used in this study. Male SD rats and C57BL/6 mice were purchased from the animal center of Guangdong Province (Guangzhou, China). The ELA^+/−^ heterozygous mice were generated by the Viewsolid Biotech Company (Beijing, China). The bodyweight of rats and mice were 250–300 g and 20–25 g respectively. All animals were cage housed with free access to tap water and standard chow, and maintained in a temperature-controlled room with 12: 12 h light–dark cycle. All the animals were randomly divided into groups as needed and euthanized with an excess intravenous dose of pentobarbital sodium (150 mg/kg, Sigma, 57-33-0) after experiments. The animal protocols were approved by the Institutional Animal Care and Use Committee of Guangzhou University of Chinese Medicine.

### Induction of hypertension and monitoring of blood pressure

Hypertension was induced in rats or mice by the subcutaneous implantation of DOCA pellets (150 mg/rat or 50 mg/mouse) 1 week after unilateral nephrectomy. One percent NaCl drinking water was provided to all the DOCA treated animals, while control animals were maintained on tap water. Rat BP was monitored via the telemetry system (Data Science International, St Paul, Minnesota, USA). Mouse BP was measured by the tail cuff method (Kent Scientific Corporation, Torrington, CT, USA).

### Intrarenal ELA overexpression

The ELA overexpression plasmid was constructed based on the commercial vector pcDNA3.1 (Invitrogen, V790-20) which carries a constitutive promoter. Briefly, the his-tag fused ELA fragment of rat was amplified with the primers CGCGGATCCATGAGATTCCAGCCCCTTTTTTG and CGGAATTCTCAATGATGATGATGATGATGGTCGACGGATGGGAAGGGCACTCGAGAAT and cloned into the BamHI-EcoRI site of pcDNA3.1, yielding the ELA overexpression plasmid pWRELAe-his. The constructed plasmid was confirmed by DNA sequencing (TsingKe Biological Technology, Beijing, China).

The intrarenal transfection of ELA in rats was performed with pWRELAe-his in combined with the transfection reagent in vivo-jetPEI (Polyplus Transfection, PT-101-01N), a polyethylenimine derivative, as previously described^31^. Briefly, the left kidney was exposed from the flank region and an interstitial infusion catheter was placed into the renal medulla, ~4–5 mm underneath the kidney surface, and secured using 3 mol/l Vet bond tissue adhesive (3M, 1469SB) and a small piece of fat tissue. Then, 50 μg of vehicle or pWRELAe-his were mixed with 8 μl of in vivo-jetPEI in 5% glucose (600 μl) and infused into the renal medulla (20 μl/min) through the infusion catheter. After infusion, the catheter was cut and blocked with fat tissue and Vetbond Tissue Adhesive.

### Assay of serum BUN, and collection of kidneys

At the endpoint of experiment, blood samples were collected from abdominal aorta while rats were anesthetized with 2% isoflurane. The serum BUN (MLBio, ml623080) and IL-1β (MLBio, ml003057) were tested by the commercial available ELISA kits. Kidneys were removed and cut longitudinally. Half of the kidney was fixed in 4% paraformaldehyde and the other half dissected into cortex and medulla, frozen in liquid N_2_, and stored at −80 °C.

### Cell culture and treatments

The human renal tubular duct epithelial cells HK2 were purchased from the Procell Life Science & Technology Company, Wuhan, China (CL-0109). The HK2 cells were grown in Dulbecco’s modified Eagle Medium F12 (Gibco, C11330500BT) supplemented with 10% (V/V) fetal bovine serum (HyClone, SV30160.03) and 1% antibiotics (Procell, PB180120) in tissue culture flasks. The cells were pretreated for 1 h with ELA peptide (1 nM, IGE Biotech, Guangzhou, China) followed by Aldosterone (0.1 µM, Sigma, A9477) treatment. After 24 h, cells were harvested for the analysis of gene expression, ROS production, and NADP^+^/NADPH.

### Morphological analysis

For morphological analysis, paraffin-embedded kidney sections (4 mm) were stained by hematoxylin–eosin. Glomerular damage was morphologically evaluated by two independent examiners who were blinded to the animal groups and semiquantitatively scored based on the degree of glomerular damage as described previously^32^. The average scores from counted glomeruli were used as the glomerular damage index for each animal.

### Quantitative reverse transcriptase PCR

For quantitative reverse transcription PCR (qRT-PCR), total RNA isolation was performed as previously described^33^. Reverse transcription and SYBR green based quantitative polymerase chain reaction were performed as the manufacturer’s instructions (TsingKe Biological Technology, TSE202). Primers used in this work are shown in Table 1.

**Table 1:** Primers used to quantify genes expression in this work.

Gene	Species	Primer sequences
ELA	Mouse	Sense: CAGAAACCAGTTAACTTTCCCAGG
		Antisense: TGGGAAGGGCACTCGAGAAT
ELA	Rat	Sense: GCGATGAGTCTCCTTTTTATCACG
		Antisense: TGGGAAGGGCACTCGAGAAT
NLRP3	Rat	Sense: CAGAAGCTGGGGTTGGTGAA
		Antisense: CCCATGTCTCCAAGGGCATT
IL-1β	Rat	Sense: TCGGCCAAGACAGGTCGCTCA
		Antisense: TGGTTGCCCATCAGAGGCAAGG
IL18	Rat	Sense: GGACTGGCTGTGACCCTATC
		Antisense: TGTGTCCTGGCACACGTTTT
MCP-1	Rat	Sense: CAGCCAGATGCAGTTAATGCC
		Antisense: AGCCGACTCATTGGGATCAT
P22phox	Rat	Sense: GCCATTGCCAGTGTGATCTA
		Antisense: CTCCTCTTCGGCCTCACTT
P47phox	Rat	Sense: CAGAATGTTGCCTGGTTG
		Antisense: GTCCCCTCCCTTAGATGA
P67phox	Rat	Sense: TCTAAGAAGCTGGCGCTCTC
		Antisense: GCGTCTGAGTTTTCCCTTTG
gp91phox	Rat	Sense: CTTCACACGGCCATTCACAC
		Antisense: GTCATAGGAGGGTTTCCGGC
GAPDH	Rat	Sense: GTCTTCACTACCATGGAGAAGG
		Antisense: TCATGGATGACCTTGGCCAG
APJ	Human	Sense: TCAGCAGCTACCTCATCTTC
		Antisense: ACTGCACCTTAGTGGTGTTC
GAPDH	Human	Sense: GATGACATCAAGAAGGTGGTG
		Antisense: GCTGTAGCCAAATTCGTTGTC

### Western blotting

Kidney tissues or cells were lysed and subsequently sonicated in PBS that contained 1% Triton X-100, 250 mmol/L phenylmethanesulfonyl fluoride, 2 mmol/L EDTA, and 5 mmol/L dithiothrietol (pH7.5). The total protein concentration was then determined by the BCA protein assay reagent kit (GBC, G3422). Thirty milligrams of protein for each sample was denatured in boiling water for 5 min then separated by SDS-PAGE and transferred onto polyvinylidene difluoride (PVDF) membranes. The blots were blocked 1 h with 5% nonfat dry milk in Tris-buffered saline, followed by incubation overnight with rabbit α-smooth muscle actin (α-SMA) antibody (Affinity, AF1032), Collagen IA (Affinity, AF7001), NLRP3 (Abcam, ab214185), Caspase-1 (Affinity, AF4005), APJ (Fitzgerald, 70R-51439), p47phox (Affinity, AF5220) or p22phox (Affinity, DF10099) at 4 °C. For β-actin, the membranes were stripped and reprobed with rabbit anti-β-actin antibody (Affinity, AF7018) or anti-GAPDH antibody (Affinity, AF7021). After being washed with Tris-buffered saline, membranes were incubated with a secondary antibody labeled with horseradish peroxidase-conjugated secondary antibody (Affinity, S0001) and visualized using enhanced chemiluminescence. The intensities of blotted bands were quantified with the software (ImageJ, free download from http://rsbweb.nih.gov/ij/).

### Immunohistochemical analysis

For immunostaining, the slides were incubated in 0.3% H_2_O_2_ diluted in 100% MeOH for 30 min to block endogenous peroxidase activity. The sections were incubated at room temperature for 30 min in PBS containing 1% BSA (Sigma, 9048-46-8) to block nonspecific binding and then incubated overnight at 4 °C in a humidified chamber with an antibody against His-tag (Affinity, T0009) or α-SMA diluted 1:500 in PBS containing 1% BSA. Then the slides were incubated for 30 min at room temperature in a humidified chamber with anti-mouse IgG antibody (Vector Laboratories, BA–9200) and then HRP conjugated streptavidin (Boster, BA1088) in PBS. Then the slides were incubated with 50 μl of diaminobenzadine (Vector Laboratories, SK4100) as a substrate, counterstained with hematoxylin (LEAGene, DH0006), dehydrated, and fixed with permount histological neutral balsam.

### Picro-sirius red staining for renal total collagen analysis

Picro-sirius red staining was used to detect collagen accumulation. The paraffin-embedded heart or kidney samples were deparaffinised with alcohol and xylene. After three washes with distilled water, renal tissues were stained in Sirius red solution for 1 h, washed in acetic acid, quickly dehydrated, and then mounted in a resinous medium. The extent of reactivity for Sirius red was quantified using image analysis software (Image-Pro Plus).

### Confocal microscopy of inflammasome proteins in HK2 cells

The cells adherent to round glass coverslips were fixed with 4% buffered paraformaldehyde and permeabilized with 0.1% Triton X-100. Then the cells were incubated with following primary antibodies: goat anti-Nlrp3 (Abcam, ab4207) and mouse anti-caspase-1 (Santa cruz, SC-56036) respectively. After incubation with primary antibodies, the dishes were washed and labeled corresponding Alexa Fluor-488 (Invitrogen, 1827671) and Alexa Fluor-555 (CST, 4409S) conjugated secondary antibodies. Then the dishes were washed and visualized through sequentially scanning on an Olympus laser scanning confocal microscope (Fluoview FV1000, Olympus, Japan). Co-localization was analyzed by Image-Pro Plus software, and the co-localization was represented by Pearson’s correlation coefficient.

### NADP+/NADPH assay kit

The NADP^+^/NADPH assay in tissues or cells was tested by the commercial kit according to the manufacture’s protocol (Beyotime Biotech, S0179). For the extraction of NADP^+^ and NADPH, the renal tissues or cells was lysed and mixed with 400 μl of extraction buffer. After centrifugation (12,000 × *g*, 10 min, 4 °C) the supernatant was collected and kept on ice in the dark. To assess the total NADP^+^/NADPH content (NADPtotal), 50 μl above supernatant of each sample was mixed with 100 μl of 10 mM G6-P and incubated for 10 min. Then the product of formazan was determined by a maximum absorbance at 450 nm (Synergy HTX multifunction, enzyme marker, Vermont, USA). To test the NADPH content, 100 μl of above supernatant of each sample was incubated in the water bath at 60 °C for 30 min since heating destroys the oxidized form of NADP (i.e., NADP+) but has no effect on the reduced form (i.e., NADPH). After heating, 50 μl supernatant of each sample was mixed with 100 μl of 10 mM G6-P and incubated for 10 min. The absorbance at 450 nm was used to determine the NADPH content. The concentration of NADPtotal and NADPH was calculated according to the standard curve respectively. The formula as followed was used to evaluate NADP^+^/NADPH Assay.

[NADP^+^] = [NADPtotal] − [NADPH],

[NADP^+^]/[NADPH] = ([NADPtotal] − [NADPH])/[NADPH].

### Reactive oxygen species ROS

The ROS production in cells was determined by a commercial kit (Nanjing Jiancheng, E004-1-1). As described in the manufacture’s protocol, the cells of each sample were incubated at 37 °C for 1 h in the serum free medium containing DCFH-DA (1:1000) (Nanjing Jiancheng, E004-1-1). Then the cells were digested by trypsin (Gibco), washed by PBS for 2–3 times, and suspended in PBS. The fluorescence signals were detected at the excitation wavelength 485 nm and the emission wavelength 525 nm (Synergy HTX multifunction).

### CRISPR/Cas9 gene editing of APJ in HK2 cells

Single-guide RNA sequence (gRNA) for CRISPR/Cas9 gene editing of protein-coding genes were designed by the CRISPR Design tool (http://crispr.mit.edu/). APJ gRNA sequences were CACCGCACAGACTGGAAATCCTCGG (sense) and AAACCCGAGGATTTCCAGTCTGTG (antisense). APJ gRNA sequences were synthesized and then inserted into the BbsI site of the plasmid pX459, generating pWHAPJg.

Gene editing in HK2 cells was carried out with Lipofectamine 3000 transfection (Invitrogen, L3000008) according to the manufacturer’s guidelines. Briefly, a master mix of pX459 or pWHAPJg was mixed with P3000 and Lipofectamine 3000 reagent in the serum free medium and incubated at room temperature for 15 min. Then the mixture was dropped into the wells and mixed softly and completely. After 24 h, the transfected cells were incubated with 3 μg/ml puromycin (Sigma, P8833) in the media to screen out the pWHAPJg‐containing cells. The gene‐editing cells were selected from diluted single clones and the APJ knockout was confirmed by PCR.

### Statistical analysis

Data are summarized as mean ± stand error (SE). Statistical analysis was performed using ANOVA followed by a Student–Newman–Keuls post hoc test for multiple comparisons or by unpaired Student *t* test for two comparisons. A *P* value < 0.05 was considered statistically significant.

## Results

### Effect of ELA overexpression on the DOCA/salt-induced hypertension

**Fig. 1:**
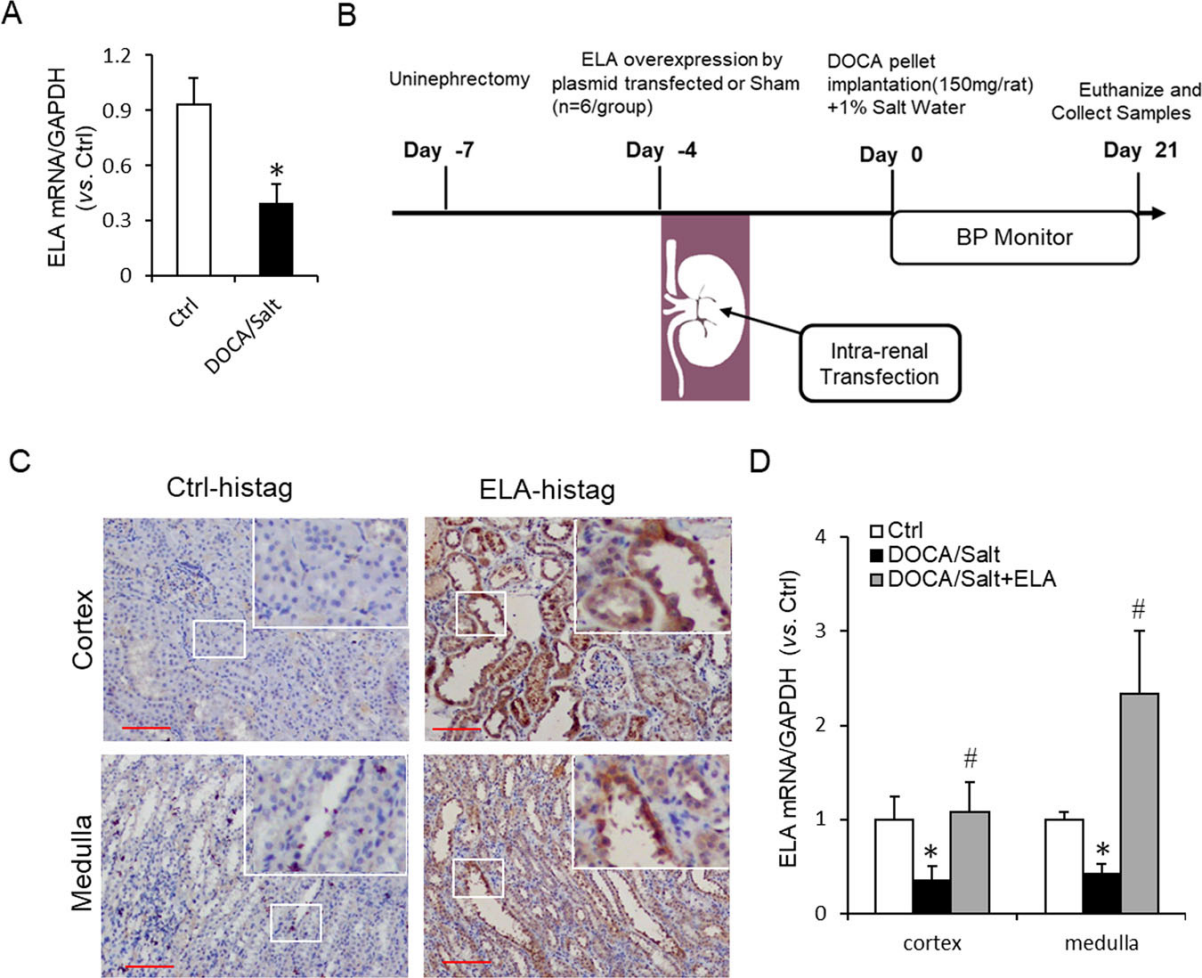
Expression and rescue experiment of ELA in DOCA/salt-induced hypertensive rats. **a** The mRNA level of ELA in the renal medulla of DOCA/salt-treated rats (*n* = 6 per group). **b** Diagram of the animal protocol for ELA treatment in this study. **c** Representative immunohistochemical staining of ELA-histag in renal cortex and medulla. Kidneys without ELA-his tag transfection were used for Negative control. Scale bar, 100 µm. **d** The mRNA level of ELA in the kidneys of control group, DOCA/salt-treated group and DOCA/salt + ELA treated group (*n* = 6 per group). *P < 0.05 versus Control group; ^#^*P* < 0.05 versus DOCA/salt group.

In DOCA/salt-treated rats, the mRNA level of ELA was found around 50% lower in the renal medulla (Fig. 1a). To explore the role of ELA on salt-sensitive hypertension, we performed intrarenal transfection of the ELA overexpression plasmid in the kidney of DOCA/salt hypertensive rats (Fig. 1b). Immunohistochemical staining for the overexpressed ELA-his tag with specific primary antibody of his-tag demonstrated an abundant accumulation of positive staining in both renal cortex and medulla in the DOCA/salt-treated rats, especially in the renal tubules (Fig. 1c). No obvious brown staining was detected in the kidneys without the ELA overexpressed vector transfection. These results verified a successful transfection and overexpression of ELA in kidney.

**Fig. 2:**
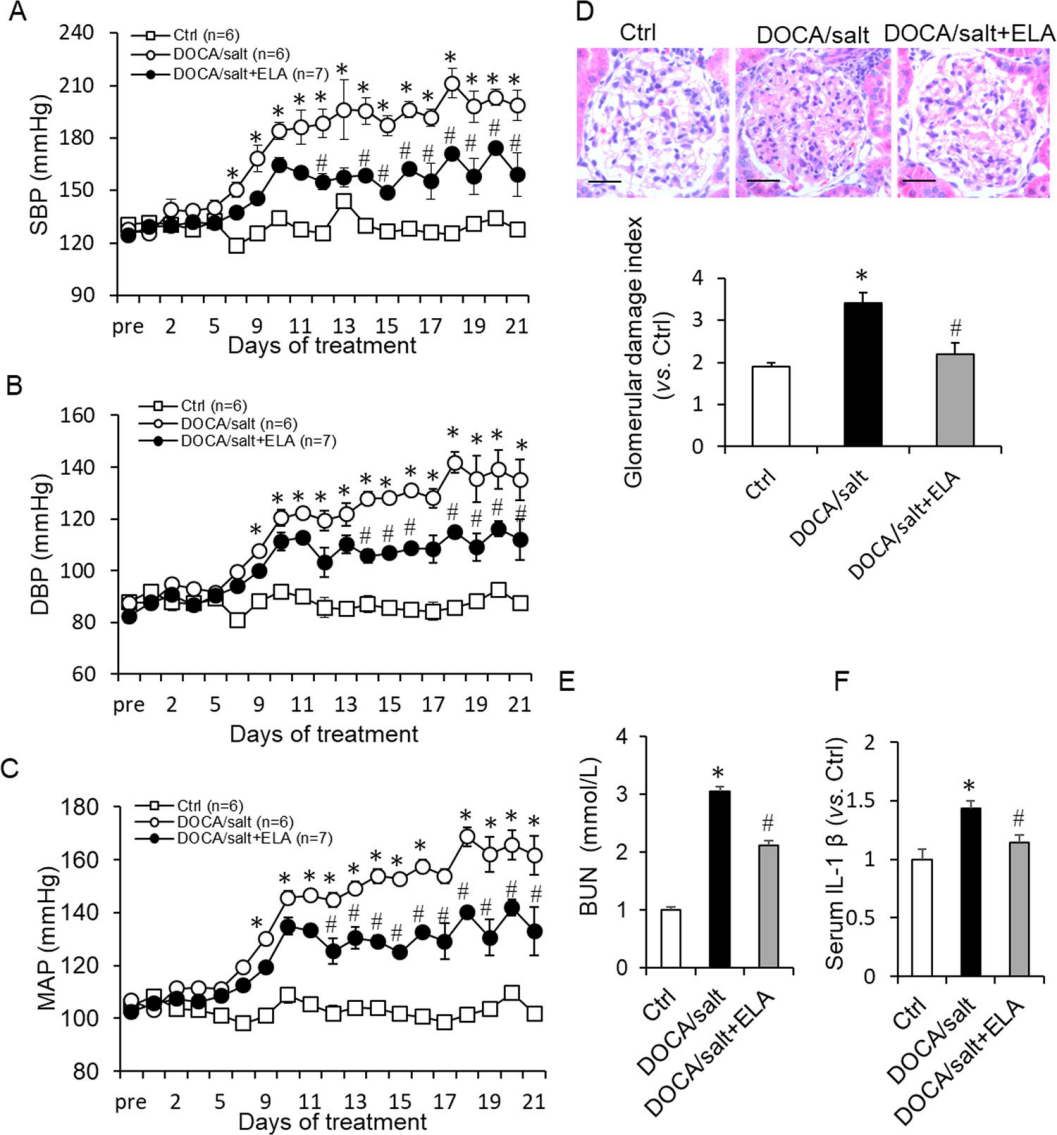
Effects of ELA on DOCA/salt-induced increase in blood pressure, serum BUN, and IL-1β. **a** Systolic blood pressure (SBP). **b** Diastolic blood pressure (DBP). **c** Mean arterial pressure (MAP). **d** Representative photomicrographs showing glomerular structures (hematoxylin–eosin staining, 400×) and summarized glomerular damage index in different groups. Scale bar, 25 µm. **e** Serum blood urea nitrogen (BUN). **f** Serum interleukin-1β (IL-1β). *N* = 6 per group. *P < 0.05 versus Control group; ^#^*P* < 0.05 versus DOCA/salt group. BUN blood urea nitrogen, IL-1β interleukin-1β.

After 3-week treatment by DOCA/salt, the DOCA/salt-suppressed mRNA level of ELA was remarkably rescued by ELA overexpression in renal cortex and medulla (Fig. 1d). As shown in Fig. 2a–c, the systolic blood pressure (SBP), diastolic blood pressure (DBP), and mean arterial pressure (MAP) were significantly increased in the DOCA/salt group. The DOCA/salt-induced increase of BP was substantially attenuated by the renal ELA overexpression but still a little higher than that of control group (Fig. 2a–c). These results demonstrated that renal ELA had an antihypertensive effect in the DOCA/salt-treated rats.

### Effects of ELA overexpression on glomerular injury and renal fibrosis in DOCA/salt-treated rats

As shown in Fig. 2d, histologic analysis showed that rats in DOCA/salt group exhibited abnormal glomerular morphology implicated by the higher glomerular damage index compared with control group. However, the morphological damage was diminished in ELA treatment group. Moreover, the DOCA/salt treatment caused a dramatic elevation of the blood urea nitrogen (BUN) levels, which was significantly attenuated by ELA treatment (Fig. 2e). Similarly, serum IL-1β induced by the DOCA/salt treatment was decreased by ELA (Fig. 2f).

**Fig. 3:**
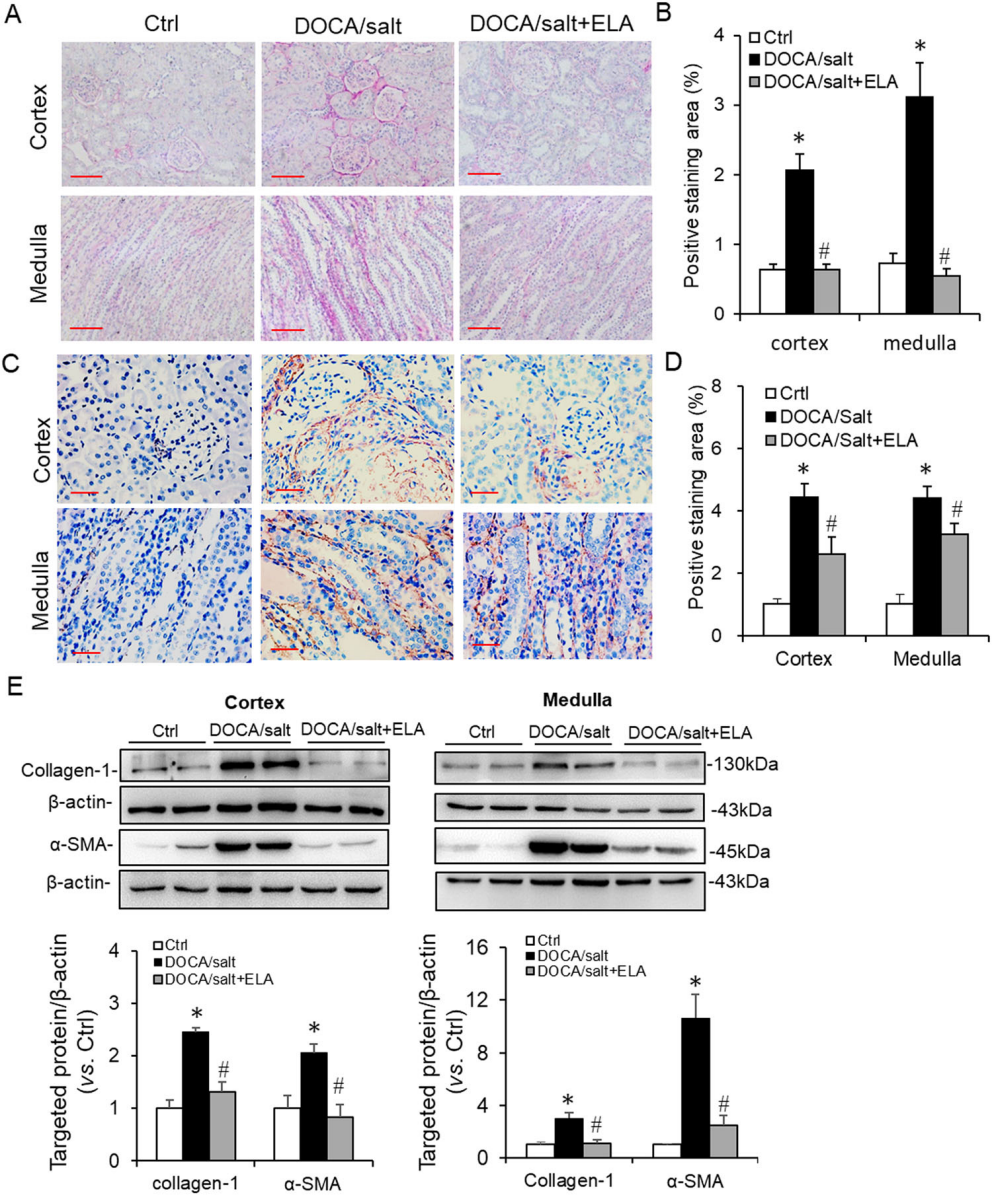
Effects of ELA on DOCA/salt-induced renal fibrosis. **a** Representative Picro-sirius red staining of collagen in renal cortex and medulla. Scale bar, 100 µm. **b** Summarized Picro-sirius red staining in renal cortex and medulla. **c** Representative immunohistochemical staining of α-SMA in renal cortex and medulla. Scale bar, 50 µm. **d** Summarized immunohistochemical staining in renal cortex and medulla. **e** Representative immunoblots of Collagen-1 and α-SMA in renal cortex and medulla as well as summarized intensities of blots. *N* = 6 per group. *P < 0.05 versus Ctrl group; ^#^*P* < 0.05 versus DOCA/salt group. α-SMA α-smooth muscle actin.

The upregulated expression of collagen and α-SMA is always used as the index of renal fibrotic injury. Thus the collagen and α-SMA levels were determined in the kidneys of these three groups. First, the Picro-sirius red was used to detect the total collagen. As shown in Fig. 3a, b, the positive-stained area of collagen was greatly enhanced in the DOCA/salt-treated kidneys than those in the control ones, which was diminished in the rats treated with ELA overexpression. Then the α-SMA level was determined by immunochemistry and the results showed that DOCA/salt remarkably increased brown staining of α-SMA in kidney but this increase was attenuated by ELA overexpression (Fig. 3c, d). Consistently, the protein levels of the collagen protein subtypes I (collagen I) and α-SMA in the renal cortex and medulla were elevated in the DOCA/salt-treated group but suppressed by ELA administration (Fig. 3e). Taken together, these data elucidated that renal ELA exerted beneficial effects on glomerular injury and renal fibrosis in DOCA/salt-induced hypertensive rats.

### Effects of ELA overexpression on the inhibition of NADPH oxidase/ROS/NLRP3 inflammasome pathway in the kidneys of DOCA/salt-induced hypertensive rats

**Fig. 4:**
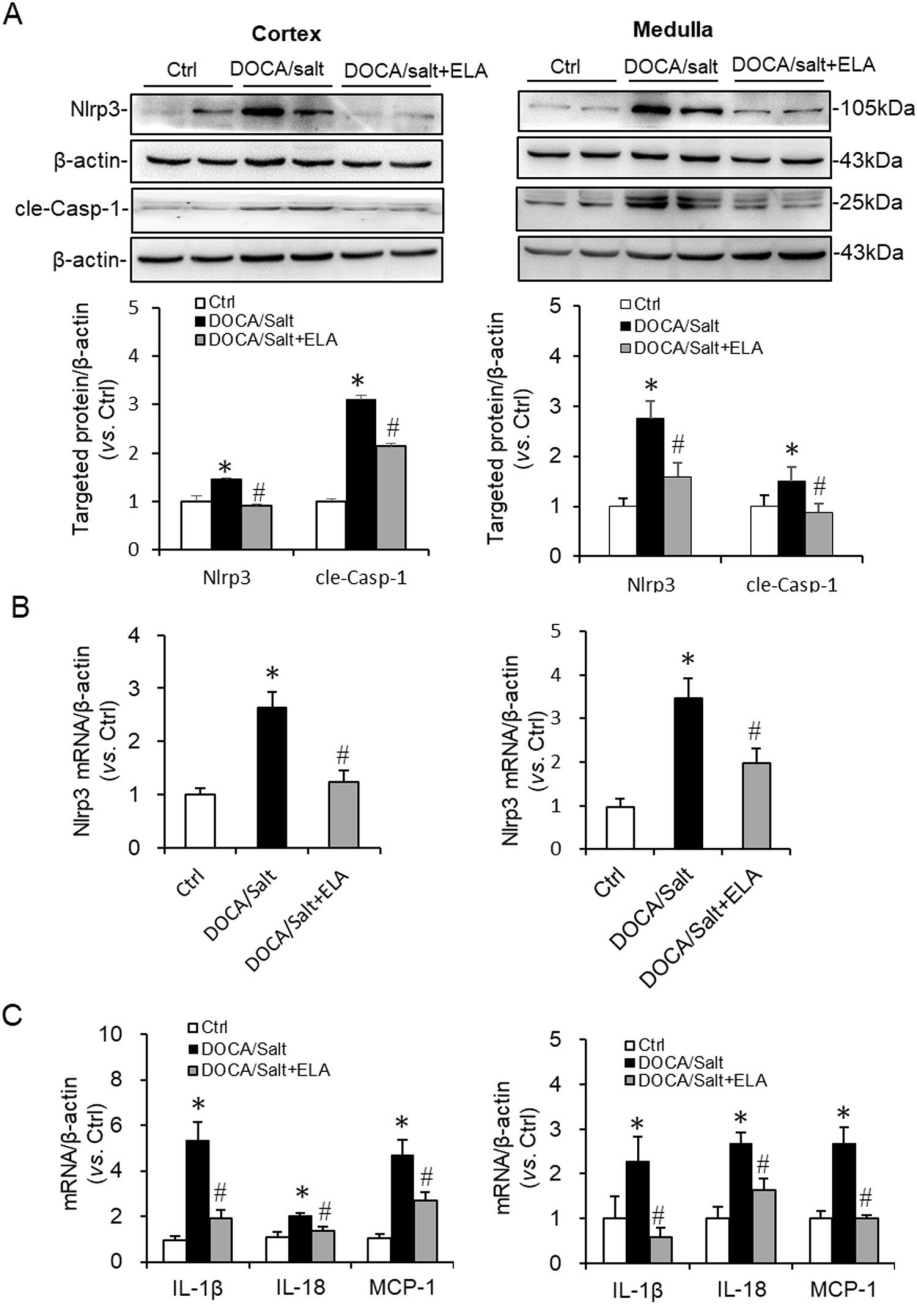
Effects of ELA on the DOCA/salt-induced activation of NLRP3 inflammasome. **a** Representative immunoblots of NLRP3 and cle-Casp-1 in renal cortex and medulla as well as summarized intensities of blots. **b** NLRP3 mRNA levels in the renal cortex and medulla. **c** mRNA levels of IL-1β, IL-18, and MCP-1 in the renal cortex and medulla. *N* = 6 per group. *P < 0.05 versus Ctrl group; ^#^*P* < 0.05 versus DOCA/Salt group. NLRP3 Nod-like receptor protein 3, cle-Casp-1 cleaved Caspase-1, IL-18 Interleukin-1β, MCP-1 monocyte chemotactic protein 1.

The mRNA and protein levels of NLRP3 in both renal cortex and medulla were significantly increased in the DOCA/salt-treated group compared to that in control group, which were dramatically inhibited by ELA (Fig. 4a, b). The same change pattern on cleaved Caspase-1 was received: compared to that in control kidneys, the increased protein level of cleaved Caspase-1 stimulated by DOCA/salt was remarkably suppressed in the DOCA/salt-ELA treated kidneys (Fig. 4a). IL-1β, IL-18, and MCP-1 are known as the downstream inflammatory markers of NLRP3 inflammasome. Thus the expression of IL-1β, IL-18, and MCP-1 were assessed. The results showed that the stimulation of these three inflammatory markers by DOCA/salt treatment was notably blunted by ELA both in the renal cortex and medulla (Fig. 4c). These data demonstrated that ELA overexpression inhibited the expression and activation of NLRP3 inflammasome in the kidneys of DOCA/salt-induced hypertensive rats.

**Fig. 5:**
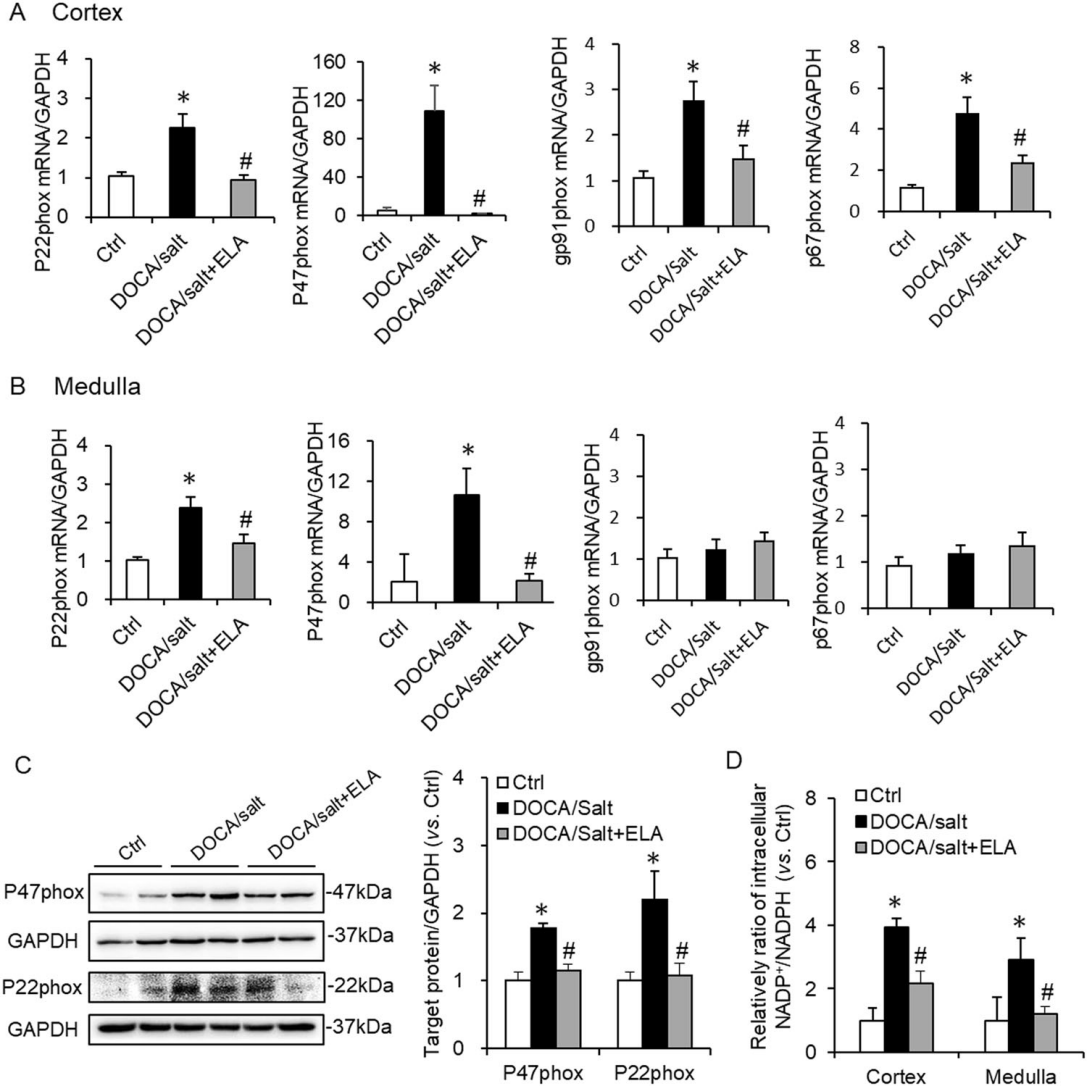
Effects of ELA on DOCA/salt-induced NADPH oxidase formation and activation. **a** mRNA levels of p22^phox^, gp91^phox^, p47^phox^, and p67^phox^ subunits of NADPH oxidase in the renal cortex. **b** mRNA levels of p22^phox^, gp91^phox^, p47^phox^, and p67^phox^ subunits of NADPH oxidase in the renal medulla. **c** Representative immunoblots of p22^phox^ and p47^phox^ and summarized intensities of blots. **d** Intracellular NADP^+^/NADPH in renal cortex and medulla. *N* = 6 per group. *P < 0.05 versus Ctrl group; ^#^*P* < 0.05 versus DOCA/salt group.

Previous studies show that NADPH oxidase donates an electron from NADPH to molecular oxygen (O_2_) to produce a superoxide anion (O_2_^−^)^34^, which is a stimulator of NLRP3 inflammasome. The activation of NADPH oxidase is dependent on the assembly of subunits, such as p22^phox^, gp91^phox^, p47^phox^, and p67^phox 17^. Hence, we determined the mRNA expression of p22^phox^, gp91^phox^, p47^phox^, and p67^phox^ in both renal cortex and medulla first. In DOCA/salt-treated group, the mRNA expression of p22^phox^, gp91^phox^, p47^phox^, and p67^phox^ were greatly elevated (Fig. 5a) in renal cortex, indicating enhanced NADPH oxidase formation. Such elevation was attenuated by ELA administration (Fig. 5a). In renal medulla, the expression patterns of p22^phox^ and p47^phox^ were similar to that in the renal cortex; however no significant expression changes were detected for gp91^phox^ and p67^phox^ in these three animal groups (Fig. 5b). Consistently, the enhanced protein levels of p22^phox^ and p47^phox^ by DOCA/salt were diminished by ELA overexpression (Fig. 5c).

Moreover, NADPH is the major subcellular provider of reducing equivalents, which can be oxidized to NADP^+^. Thus the ratio of NADP^+^ to NADPH (NADP^+^/NADPH) is usually used to reflect the NADPH oxidase activation and subsequently ROS production^35^. As shown in Fig. 5d, the ratio of NADP^+^/NADPH was significantly increased in DOCA/salt-treated group, indicating the activation of NADPH oxidase and raised ROS production. And such increase was obviously alleviated by ELA in kidney.

Taken together, the data above suggested that renal ELA exerted suppressing effects on the NADPH oxidase/ROS/NLRP3 inflammasome pathway in the DOCA/salt-induced hypertension.

### Effects of ELA on the NADPH oxidase/ROS/NLRP3 inflammasome pathway induced by Aldosterone in HK2 cells

**Fig. 6:**
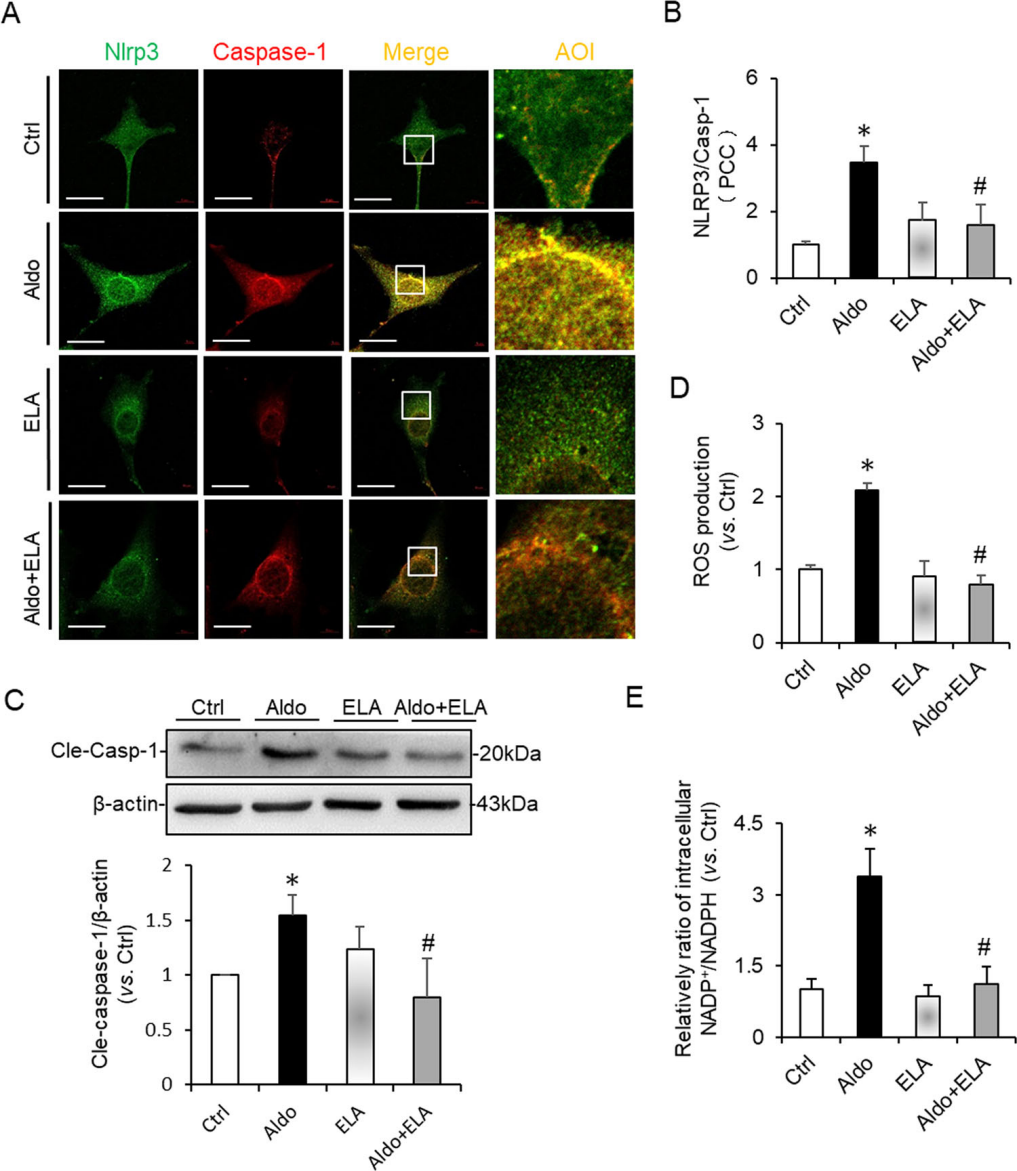
Effects of ELA on Aldosterone-induced NADPH oxidase/ROS/NLRP3 pathway in HK2 cells. The HK2 cells were pretreated with ELA peptide (1 nM) for 1 h and then treated by Aldosterone (0.1 µM) for 24 h. **a**, **b** representative confocal fluorescent images of co-localization (yellow) between Nlrp3 (green)/Caspase-1 (red), Nlrp3 (green)/ASC (red) and summarized co-localization coefficient (*n* = 5 per group). Scale bar, 10 µm. **c** Representative immunoblots of cleaved Caspase-1 and summarized intensities of blots (*n* = 5 per group). **d** ROS production (*n* = 6 per group). **e** Intracellular NADP^+^/NADPH (*n* = 6 per group). *P < 0.05 versus Ctrl group; ^#^*P* < 0.05 versus DOCA/salt group. ROS reactive oxygen species, NADPH nicotinamide adenine dinucleotide phosphate.

To further confirm the suppressing effects of ELA on the NADPH oxidase/ROS/NLRP3 inflammasome pathway, a series of tests were assessed in HK2 cells after the treatment of Aldosterone (Aldo) with or without ELA peptide. First, we determined the effect of ELA on the formation and activation of NLRP3 inflammasome induced by Aldo in HK2 cells. Aldo treatment resulted in strengthened co-localization (yellow spots) of NLRP3 molecules (green) with Caspase-1 (red) in HK2 cells compared to that of control cells, indicating enhanced formation of NLRP3 inflammasome (Fig. 6a). Unsurprisingly, such strengthened co-localization was significantly attenuated by ELA peptide (Fig. 6a). The co-localization coefficient analyses were summarized in Fig. 6b. Consistently, Aldo-induced Caspase-1 cleavage was inhibited by ELA (Fig. 6c). Furthermore, the increased ROS production (Fig. 6d) and the enhanced ratio of NADP+/NADPH (Fig. 6e) were observed in Aldo treated cells, which were markedly alleviated by ELA pretreatment. No significant difference was detected on the co-localization of NLRP3 with caspase-1, the protein level of cleaved Caspase-1, ROS production and the ratio of NADP+/NADPH in cells only treated with ELA treatment or not. These results clearly suggested that ELA had inhibitory effects on the Aldosterone-induced NADPH oxidase/ROS/NLRP3 inflammasome in HK2 cells.

### Effects of ELA in the DOCA/salt-induced hypertension are APJ independent

**Fig. 7:**
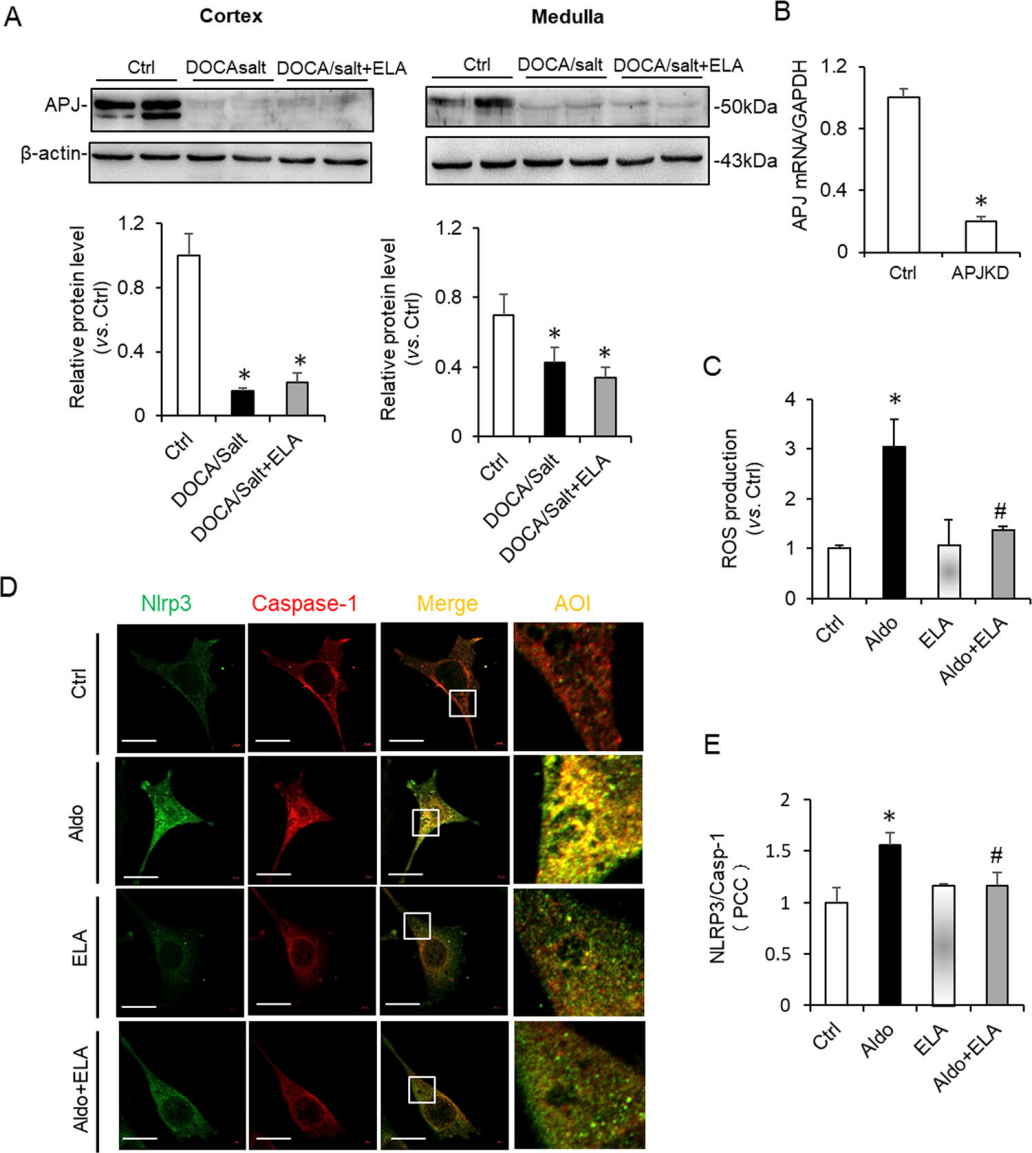
Effects of ELA on the ROS production and NLRP3 imflammasome activation are independent of APJ. APJ knockdown was conducted by the transfection of plasmid pWHAPJg into HK2 cells and confirmed by RT-PCR. Then the HK2 cells with APJ knockdown were pretreated with ELA peptide (1 nM) for 1 h and then treated by Aldosterone (0.1 µM) for 24 h. **a** Representative immunoblots of APJ in renal cortex and medulla and summarized intensities of blots. **b** mRNA levels of APJ in HK2 cells with or without APJ knockdown. **c** ROS production in HK2 cells with APJ knockdown. **d**, **e** Representative confocal fluorescent images of co-localization (yellow) between Nlrp3 (green)/Caspase-1 (red) and summarized co-localization coefficient in HK2 cells with APJ knockdown. Scale bar, 10 µm. *N* = 6 per group. *P < 0.05 versus Ctrl group; ^#^*P* < 0.05 versus DOCA/salt group.

ELA is reported to be an endogenous ligand for apelin receptor (APJ) and regulates fluid homeostasis by binding to APJ in kidney^26^. And APJ has been found involved in the regulation of NADPH oxidase and ROS production^36,37^. Thus, we examined whether ELA was dependent on APJ to inhibit NADPH oxidase/ROS/NLRP3 inflammasome pathway in the DOCA/salt-treated kidneys. As shown in Fig. 7a, the protein level of APJ was dramatically inhibited both in renal cortex and medulla of the DOCA/salt-treated rats. ELA overexpression was unable to recover the inhibition of APJ by DOCA/salt (Fig. 7a), implying that the antihypertensive effect of ELA may be independent on APJ in vivo. To further investigate whether APJ is involved in the inhibitory effects of ELA on Aldo-induced NADPH oxidase/ROS/NLRP3 inflammasome pathway, APJ was knocked down (KD) in HK2 cells, which was verified by real-time PCR (Fig. 7b). Interestingly, Aldo-induced ROS production was still suppressed by ELA in the APJ KD cells (Fig. 7c). Consistently, ELA peptides blocked the enhanced co-localization (yellow spots) of NLRP3 molecules (green) with caspase-1 (red) stimulated by Aldo in APJ KD cells (Fig. 7d). The co-localization coefficient analyses were summarized in Fig. 7e. No statistical significance was detected on the ROS production or co-localization of NLRP3 molecules (green) with caspase-1 (red) in APJ KD cells only treated with ELA treatment or not. Thus, our in vivo and in vitro results suggested that the protective roles of ELA in the DOCA/salt-induced hypertension are independent on APJ in the kidney.

### ELA deficiency accelerated the onset of hypertension in DOCA/salt hypertensive mice

**Fig. 8:**
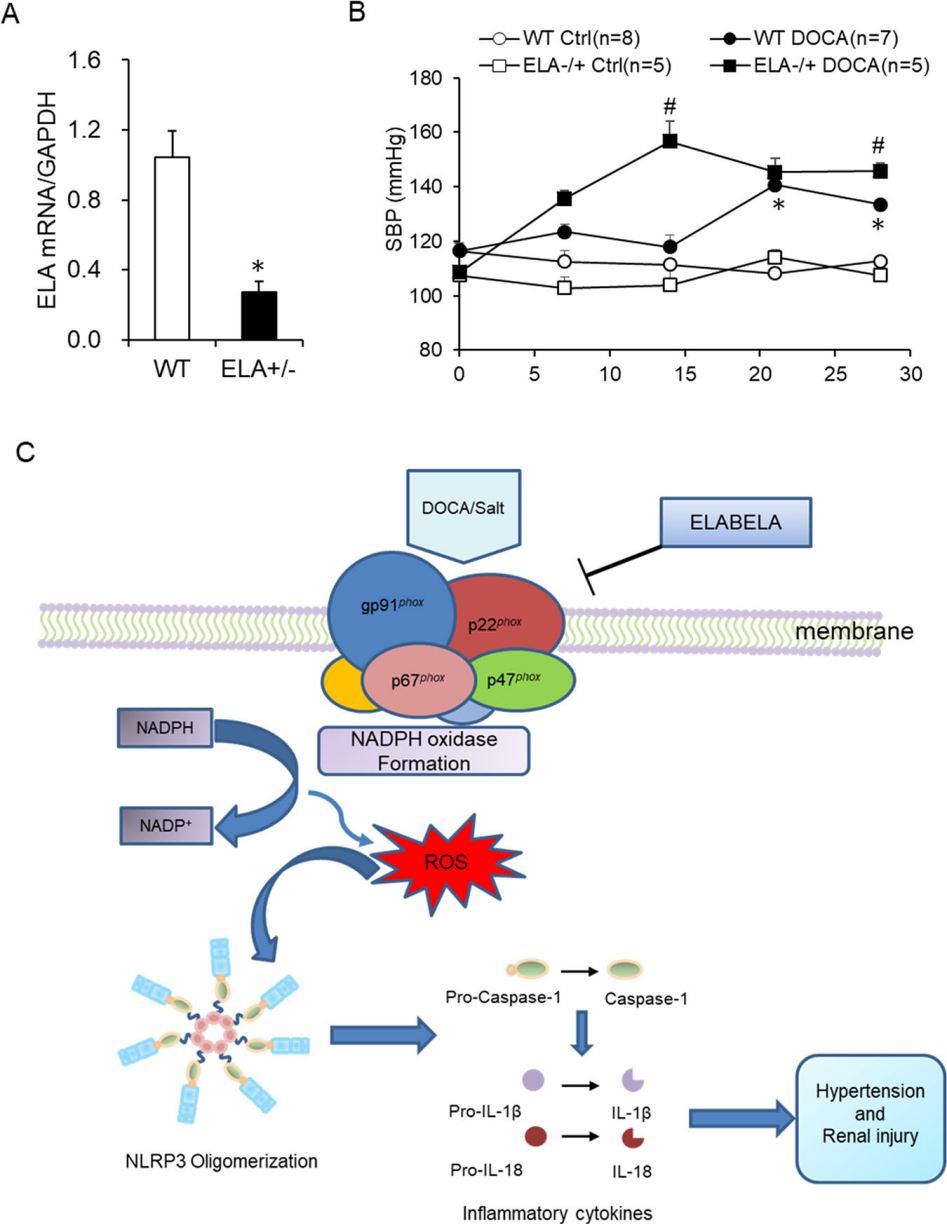
Effects of ELA deficiency on DOCA/salt-induced hypertension and potential mechanism of ELA exerted on DOCA/salt-induced hypertension. **a** The mRNA level of ELA in the kidney of heterozygous ELA knockout C57BL/6 mice (ELA^+/−^) compared to WT ones. *N* = 5. **b** Systolic blood pressure (SBP). **c** Potential mechanism of ELA exerted on DOCA/salt-induced hypertension. In DOCA/salt-treated kidney, ELA blocks the NADPH oxidase formation by inhibiting the expression of its subunits p22^phox^, gp91^phox^, p47^phox^, and p67^phox^; decreases the ROS production; obstructs the recruitment and activation of NLRP3 inflammasome; attenuates hypertension and renal injury. *P < 0.05 versus WT Ctrl group. ^#^*P* < 0.05 versus DOCA/salt-treated WT group.

To further confirm the antihypertensive effect of ELA, the ELA^+/−^ mice were used to observe the change of BP with DOCA/salt treatment. The mRNA expression of ELA was about 70% decreased in the kidney of the ELA^+/−^ mice compared to that of WT mice (Fig. 8a), suggesting a successful knockdown of ELA. As shown in Fig. 8b, DOCA/salt treatment progressively increased the SBP in the ELA^+/−^ and WT mice, while the BP remained unchanged in the WT control and ELA^+/−^ control animals. More interestingly, compared to WT mice, ELA^+/−^ mice exhibited an earlier increase of SBP in response to the DOCA/salt treatment, especially in the first 2 weeks (Fig. 8b). The SBP of ELA^+/−^ and WT mice treated with DOCA/salt at the 14th day were 156 ± 7 mmHg and 120 ± 4 mmHg respectively. At the end of the animal experiment, the SBP of the ELA^+/−^ DOCA/salt group (146 ± 3 mmHg) was still a little higher than that of the WT DOCA/salt group (134 ± 2 mmHg) but no statistical significance existed between WT control group and ELA^+/−^ control group (112 ± 2 mmHg vs. 108 ± 2 mmHg). These results above suggested that ELA deficiency dramatically accelerated the onset of hypertension in DOCA/salt-treated mice, supporting a protective role of ELA on salt-sensitive hypertension.

## Discussion

In the present study, we investigated the roles of ELA in DOCA/salt-induced hypertension and the potential mechanism. In DOCA/salt-treated rats, the mRNA level of ELA greatly decreased in the renal medulla (Fig. 1a). Then we found that overexpression of ELA in the kidney attenuated DOCA/salt-induced hypertension and renal injury, including lower BP, reversed glomerular morphological change, decreased serum BUN, and blocked fibrotic accumulation (Figs. 2, 3). Mechanistically, ELA overexpression inhibited renal NADPH oxidase activity and subsequent ROS production, thus resulted in the blockade of formation and activation of NLRP3 inflammasome stimulated by DOCA/salt (Figs. 4, 5). The inhibitory effects of ELA on Aldosterone-stimulated NADPH oxidase/ROS/NLRP3 inflammasome signaling pathway was further confirmed in the HK2 cells (Fig. 6). Furthermore, our in vivo and in vitro results showed that the deficiency of the apelin receptor APJ did not influence on the antihypertensive effect and blockage to NADPH oxidase/ROS/NLRP3 pathway of ELA (Fig. 7). Additionally, in heterozygous ELA knockout mice (ELA^+/−^), we found that ELA deficiency significantly accelerated the onset of DOCA/salt-induced hypertension (Fig. 8a, b). Taken together, our data suggest that renal ELA exerts its beneficial roles in DOCA/salt-induced hypertension via blocking NADPH oxidase/ROS/NLRP3 inflammasome pathway, which is independent on its endogenous receptor APJ. The potential mechanism of ELA functions on DOCA/salt-induced hypertension and renal injury was shown in Fig. 8c.

Previous studies show that ELA is expressed not only in embryos but also in adult kidneys^24,29^. In humans, a 54-amino-acid (aa) full length of ELA bears a cleavage to yield a secretory mature form comprised of 32 aa^23^. The amino-acid sequence analysis reveals multiple dibasic sites in the 32-aa ELA (ELA-32), implying more shorten isoforms existing in vivo^38^. Among these isoforms, an 11-aa ELA (ELA-11) is highly conserved and endogenously observed via mass spectrometry in embryos^25^. It has been recently reported that both ELA-32 and ELA-11 are able to significantly inhibit H/R-induced renal fibrosis, inflammation, renal tubular lesions, and renal dysfunction^27^. They also report that ELA-11 but not ELA-32 has inhibitory effects on I/R-induced autophagy in the kidney. It implies that different isoforms of ELA may function under different mechanisms. Whether there is a major functional form existing is not yet known. In this work, we tried to overexpress ELA peptide in the kidney by a transfected plasmid. The synthesized full length of ELA may be cleaved into various active forms and secreted into the whole kidney. The immunohistochemical results showed that ELA peptides existed in both renal cortex and medulla, especially in the renal tubules (Fig. 2b), which is consistent to the previous studies^27,28^. However, there are still some limits in this work. First, since lacking commercial and specific antibody of ELA, the protein level couldn’t be determined in kidneys under all the experimental conditions. On the other hand, our results cannot tell different isoforms of ELA locally since the his tag was fused to the C terminus of ELA peptide.

Oxidative stress and inflammation are found to play essential roles in the occurrence and development of cardiaovascular diseases, chronic kidney diseases, diabetes with microvascular complications, tumors, and preeclampsia^39–42^. Documents of studies show that ELA endogenous homologue apelin, through binding to its receptor APJ, is involved in the regulation of oxidative stress and inflammation^43^. However, whether the role of apelin is beneficial or aggravating remains controversial. For example, a previous study demonstrates that apelin reduces oxidative stress and prevents pressure‐overload‐induced left ventricular hypertrophy^44^. By contrast, there is increasing evidence proving that apelin-13 induces cardiac hypertrophy by increasing the production of ROS and the expression levels of NADPH oxidases in vivo and in vitro^45–47^. In addition, apelin/APJ in paraventricular nucleus is found to induce long-term high BP and renal sympathetic nerve activity via increasing oxidative stress^37,48^. Thus, there is no identical conclusion on the association of apelin/APJ with oxidative stress and inflammation. Different from that on apelin, the related studies on ELA are limited. Our data demonstrated that ELA had inhibitory effects on NADPH oxidase/ROS/NLRP3 pathway in the kidney to improve DOCA/salt-induced hypertension. Such effects of ELA peptide were also confirmed in the human renal tubular duct epithelial cells.

In addition, it is known that the regulatory effects of apelin on oxidative stress are conducted by binding to its receptor APJ in most experimental conditions^37,46,47^. As another endogenous ligand, it is still unclear whether ELA is dependent on APJ to suppress DOCA/salt-induced NADPH oxidase/ROS/NLRP3 signaling pathway. Our results in this work found that APJ was dramatically inhibited in both renal cortex and medulla in DOCA/salt-induced hypertensive rats and such inhibition was not rescued by overexpression of ELA. These indicated that APJ may have no effect on the improving roles of ELA in the DOCA/salt-treated kidney. Moreover, even as APJ was around 80% downregulated in HK2 cells, ELA still blocked the Aldo-induced ROS production and NLRP3 inflammasome formation. Thus, our results suggested that the antihypertensive effect and inhibition to NADPH oxidase/ROS/NLRP3 pathway of ELA does not rely on the G protein receptor APJ. It further raised an interesting question how ELA works on NADPH oxidase/ROS in DOCA/salt-treated rats, directly or indirectly. Similar phenomena are reported recently that ELA can function independently on APJ^27^, although ELA is verified to directly bind to the APJ receptor with high affinity^26^. Little is known about the underlying mechanism. It is assumed by Chen et al. that higher dosages of ELA (0.3 and 3 mM) but not lower dose (300 pM) could induce APJ endocytosis and the downstream phosphorylation of ERK to accomplish the reno-protective functions^27^. There is another possibility that other kind of receptor may exist to mediate the effects of ELA. Thus, it is of great interest to explore whether other potential receptors or a competitive mechanism is involved for further understanding the roles of ELA in the kidney.

There are several studies showing that exogenous treatment of ELA peptides results in protective and improving effects on kidney. Deng et al. find that intravenous injections of ELA peptide increase the urine flow rate and water intake and these effects are dose dependent^26^. Another study suggests that ELA treatment protects the kidney from I/R stress^27^. To fully understand the roles of ELA, the ELA knockout mouse is a useful tool. However, a recent study reveals that knockout of ELA leads to lethal cardiac defects in some ELA null embryos^49^. Thus, heterozygous ELA knockout C57BL/6 mice (ELA^+/−^) were used to verify its functions in this work. Compared to the WT mice, the expression of ELA in ELA^+/−^ kidneys was about 70% lower (Fig. 8a), suggesting a successful ELA deficiency. We found that the ELA^+/−^ mice presented no significant hypertension at the basic condition (Fig. 8b). However, ELA^+/−^ mice exhibited an earlier increase of SBP than WT ones in response to the DOCA/salt treatment, especially in the first 2 weeks (Fig. 8b). At the end of the animal experiment, the ELA^+/−^ DOCA/salt group still had a little higher of SBP than the WT DOCA/salt group. These results of BP suggested that ELA deficiency dramatically accelerated the onset of hypertension in DOCA/salt-induced hypertensive mice, further confirming a protective role of ELA on salt-sensitive hypertension.

## Conclusion

In summary, the present study investigates the role of ELA in the development of DOCA/salt-induced hypertension and renal injury in rodent models. Our data demonstrate that ELA may prevent DOCA/salt-induced hypertension by blocking NADPH oxidase/ROS/NLRP3 signaling pathway in the kidney, which is APJ independent. All these provide new sight on ELA as a candidate to treat salt-sensitive hypertension and renal injury.
